# Molecular Characterization of Predominant Serotypes, Drug Resistance, and Virulence Genes of *Streptococcus pneumoniae* Isolates From East China

**DOI:** 10.3389/fmicb.2022.892364

**Published:** 2022-06-01

**Authors:** Li-Dan Huang, Mei-Juan Yang, Yan-Ying Huang, Ke-Yi Jiang, Jie Yan, Ai-Hua Sun

**Affiliations:** ^1^School of Basic Medical Sciences and Forensic Medicine, Hangzhou Medical College, Hangzhou, China; ^2^Hangzhou Chest Hospital, Zhejiang University School of Medicine, Hangzhou, China; ^3^Department of Medical Microbiology and Parasitology, Zhejiang University School of Medicine, Hangzhou, China

**Keywords:** East China, *Streptococcus pneumoniae*, predominant serotypes, drug resistance, virulence genes

## Abstract

*Streptococcus pneumoniae* is a common diplococcus pathogen found worldwide. The characterization of predominant serotypes, drug resistance, and virulence genes of *S. pneumoniae* isolates prevailing in different areas and countries is clinically important for choice of antibiotics and improvement of vaccines. In this study, pneumonia (78.7%) and meningitis (37.0%) were the predominant diseases observed in the 282 (children) and 27 (adults) *S. pneumoniae*-infected patients (*p* < 0.05) from seven hospitals in different areas of East China. Of the 309 pneumococcal isolates, 90.3% were classified by PCR into 15 serotypes, with serotypes 19F (27.2%) and the 6A/B (19.1%) being most predominant (*p* < 0.05). Importantly, serotypes 15A and 15B/C combined for a total of 10.4% of the isolates, but these serotypes are not included in the 13-valent pneumococcal capsule conjugate vaccine used in China. Antimicrobial susceptibility analysis by the *E*-test showed that >95% of the 309 pneumococcal isolates were susceptible to moxifloxacin and levofloxacin, as well as 18.4, 85.8, and 81.6% of the isolates displayed susceptibility to penicillin, cefotaxime, and imipenem, respectively. A significant correlation between the prevalence of predominant serotypes and their penicillin resistance was observed (*p* < 0.05). In particular, >95% of all the pneumococcal isolates showed resistance to erythromycin and azithromycin. Of the nine detected virulence genes, the *lytA*, *ply*, *hysA*, and *nanA* were the most common with 95–100% positive rates in the 309 pneumococcal isolates, while the *pavA* and *psaA* genes displayed a significant correlation with pneumococcal bacteremia and meningitis (*p* < 0.05). Overall, our data suggested that the predominant serotypes, drug resistance, and virulence genes of the *S. pneumoniae* isolates prevailing in East China are distinct from those observed in other areas of China and adjacent countries.

## Introduction

*Streptococcus pneumoniae* is a common Gram-positive diplococcus pathogen that mainly causes pneumonia, upper respiratory tract infections (URTIs), and tympanitis in children (Dockrell et al., 2012; Korona-Glowniak et al., 2018; Oligbu et al., 2019). In the recent decade, *S. pneumoniae* has been frequently reported as the causative agent of community-acquired pneumonia, bacteremia, and meningitis in adults (Rozenbaum et al., 2013; Argemi et al., 2016; Oordt-Speets et al., 2018). However, the incidence of pneumococcal diseases in developing countries is significantly higher than in developed countries due to different economic and medical conditions (Amin et al., 2014; Droz et al., 2019).

Vaccination is the crucial measure to prevent from infections by microbial pathogens including *S. pneumoniae*. At present, the mainly used pneumococcal vaccine used in China is a 13-valent pneumococcal capsule conjugate containing the predominant or common serotypes of *S. pneumoniae* prevailing in China (Wang et al., 2021). However, the predominant serotypes of *S. pneumoniae* in different areas and countries are usually distinct and changed continually (Kim et al., 2020). Therefore, regular investigation of the predominant *S. pneumoniae* serotypes present in the different areas of China is necessary for control of pneumococcal diseases and improvement of pneumococcal vaccines.

β-Lactam antibiotics such as penicillin (PNC), cefotaxime (CTX), and imipenem (IPM), macrolide antibiotics such as erythromycin (ETM) and azithromycin (AZM), and quinolone antibiotics such as levofloxacin (LEV) and moxifloxacin (MXF) are generally used for treatment of pneumococcal diseases in clinics. Many epidemiological studies revealed that *S. pneumoniae* isolates from patients frequently display single or multiple drug resistance against different classes of antibiotics. *S. pneumoniae* drug resistance not only complicates antimicrobial therapy of pneumococcal diseases but also results in the specific prevalence of drug-resistant *S. pneumoniae* serotypes (Thummeepak et al., 2015; Nakano et al., 2016; Yahiaoui et al., 2016; Aliberti et al., 2019; Suaya et al., 2020). However, the drug resistance spectrum of *S. pneumoniae* isolates from different areas of China and the correlation between drug resistance and prevalence of different *S. pneumoniae* serotypes remain poorly characterized.

The *S. pneumoniae* capsule is its most important virulence factor because capsule-null strains are severely attenuated for their ability to cause any diseases in human and animals (van der Poll and Opal, 2009). Previous studies demonstrated that the capsule of *S. pneumonias* is able to resist the phagocytosis by phagocytes and promotes the pneumococcal colonization of host cells (Nelson et al., 2007; Brissac et al., 2021). Moreover, the capsule is the main surface antigen of *S. pneumoniae* and the basis for pneumococcal serotype classification (Weinberger et al., 2009). Except for capsule, *S. pneumoniae* can produce many other virulence factors for adherence, colonization, and invasion of hosts (Subramanian et al., 2019). For example, the products of the *S. pneumoniae cbpA* and *2A* genes serve as an adherence factor and a component in the capsule, respectively (Zhao et al., 2019). The hyaluronidase expressed by the *S. pneumoniae hysA* gene is utilized for invasion into tissues of hosts (Marion et al., 2012). The autolysin, expressed by the *S. pneumoniae lytA* gene, is toxic due to the release of cell wall degradation products, which evokes inflammation reactions in hosts and Ply pneumolysin to induce DNA damage of cells (Mellro et al., 2014; Rai et al., 2016). The pneumococcal NanA neuraminidase promotes the formation of pneumococcal biofilms and facilitates the colonization by *S. pneumoniae* (Wren et al., 2017). PavA, a fibronectin-binding protein, mediates pneumococcal adherence to endothelial cells, while the PsaA, an adherence factor, is essential for the virulence of *S. pneumoniae* (Holmes et al., 2001; Romero-Steiner et al., 2003). Surface protein A, the product of the *S. pneumoniae pspA* gene, can inhibit complement-mediated phagocytosis after binding to lactoferrin (Croney et al., 2013). However, the correlation between these virulence factors and predominant serotypes of *S. pneumoniae* has not been well characterized.

In this study, 309 *S. pneumoniae* isolates collected from the seven hospitals in different areas in East China during 2021 were used for serotype classification, drug resistance detection, and virulence factor examination. The aim of this study was to understand the diversity of predominant pneumococcal diseases in children and adults, reveal the predominant pneumococcal serotypes prevailing in East China, and determine the correlations between predominant or common pneumococcal serotypes with their drug resistance and virulence factors. These results will be helpful for antibiotic choice during treatment of pneumococcal diseases and improvement of pneumococcal capsule conjugate vaccines by including capsular polysaccharide antigens from novel prevailing *S. pneumoniae* serotypes.

## Materials and Methods

### Separation and Identification of *S. pneumoniae* Isolates

Sputum, bronchoalveolar lavage fluid, peripheral blood, and cerebrospinal fluid samples of 4,665 patients suffering from bacterial URTI, pneumonia, tympanitis, bacteremia, or meningitis according to clinical signs and symptoms and laboratory examinations were collected in the year 2021 from the seven hospitals located in different areas of East China for bacterial isolated cultivation and identification. Briefly, each of the samples was inoculated on a Columbia blood agar plate (bioMérieux, France) and then incubated at 37°C with 5% CO_2_ for 24 h. Individual bacterial colonies grown on the agar plates were identified using the VITEK 2-Compact Auto-Bacterial Detector (bioMérieux). In this study, 309 cases of the patients’ samples (282 children and 27 adults) were isolated for *S. pneumoniae*, and all the 309 pneumococcal isolates were used. Besides, the *S. pneumoniae* isolates were confirmed again using the colony morphological examination, Gram staining, optochin test, and bile lysis test before use. In the examinations, staining, and tests, *S. pneumoniae* ATCC49619 was used as the control.

### β-Lactamase Phenotype Confirmatory Test

β-Lactam antibiotics are the most commonly used antibacterial drugs in clinics. β-Lactamases play a critical role in bacterial resistance to β-lactam antibiotics. In this study, a β-lactamase phenotype confirmatory test was performed to detect the possible β-lactamases produced by the *S. pneumoniae* isolates using a Cephalothin Disk Kit (Oxoid, England) (Khan et al., 2014). The cephalothin disk became red rapidly within 5 min when a bacterial strain produced a β-lactamase. In the test, *Staphylococcus aureus* ATCC29213, *S. aureus* ATCC25923, and *S. pneumoniae* ATCC49619 were used as the positive and negative controls, respectively.

### Serotyping of *S. pneumoniae* Isolates

The serotypes of 309 *S. pneumoniae* isolates were identified using PCR as previously reported (Pai et al., 2006; Dias et al., 2007; Da Gloria-Carvalho et al., 2010). Briefly, genomic DNA of the pneumococcal isolates was extracted using a Bacterial Genomic DNA MiniPrep Kit (Axygen, United States) and then quantified using ultraviolet spectrophotometry (Sun et al., 2010). Using 100 ng DNA of each of the genomic DNA samples as the template, separate PCRs were performed using a High-Fidelity Ex-Taq PCR Kit (TaKaRa, China) and deferent pairs of serotyping primers (Supplementary Table 1). The PCRs were initiated by incubation at 94°C for 5 min for DNA denaturation, followed by 35 cycles at 94°C for 30 s, 52–54°C for 30 s, and 72°C for 30–60 s to amplify the target gene fragments and finally an incubation step at 72°C for 7 min. The *S. pneumoniae* 16S rRNA gene and *cpsA* gene, a sequence-conserved capsule formation-determining gene, were used as the controls (El Aila et al., 2010; Coskun-Ari et al., 2012). For each of the genomic DNA samples, three independent PCR repeats were performed for serotype detection.

### Drug Susceptibility Test

An *E*-test was used to detect the minimum inhibitory concentration (MIC) of PNC, CTX, IPM, ETM, AZM, LEV, and MXF for the 309 *S. pneumoniae* isolates. The obtained results were defined according to the 2021 Clinical and Laboratory Standards Institute (CLSI) Guideline M100-Ed31. Strains such as *S. aureus* ATCC25923, *Escherichia coli* ATCC25922, and *S. pneumoniae* ATCC49619 were used as the controls.

### Detection of Virulence Genes in *S. pneumoniae* Isolates

Although *S. pneumoniae* does not produce endotoxin [i.e., lipopolysaccharide (LPS)] and typical exotoxins, this pathogenic diplococcus has many other virulence genes in its genome, such as the *cbpA*, *cps2A*, *hysA*, *lytA*, *nanA*, *pavA*, *ply*, *psaA*, and *pspA* genes, which encode products important for adherence, colonization, invasion, and survival (Holmes et al., 2001; Romero-Steiner et al., 2003; Marion et al., 2012; Croney et al., 2013; Mellro et al., 2014; Rai et al., 2016; Wren et al., 2017; Subramanian et al., 2019; Zhao et al., 2019). The presence of these nine virulence genes in each of the 309 *S. pneumoniae* isolates was detected by individual PCRs using a High-Fidelity Ex-Taq PCR Kit (TaKaRa) and deferent pairs of primers (Supplementary Table 2). The PCRs were initiated by incubation at 94°C for 5 min for DNA denaturation, followed by 35 cycles at 94°C for 30 s, 52–54°C for 30 s, and 72°C for 30–60 s to amplify the target gene fragments, and then incubation at 72°C for 7 min for extension. The 16S rRNA gene was used as the control (El Aila et al., 2010). For each of the virulence genes in the *S. pneumoniae* isolates, three independent PCR repeats were performed for detection.

### Statistical Analysis

The obtained data were statistically analyzed using the SPSS 20.0 software. Chi-square test and Fisher’s exact probability examination were used to determine significant differences. The statistical significance was defined as *p* < 0.05.

## Results

### Predominant Diseases in *S. pneumoniae*-Infected Children and Adults

Of the samples collected from 4,665 patients suffering from bacterial URTI, pneumonia, tympanitis, bacteremia, or meningitis according to clinical signs and symptoms and laboratory examinations, a total of 309 cases were isolated for *S. pneumoniae*. Of the 309 patients infected with *S. pneumoniae*, 91.3% (282/309) were children (≤12 years old), while 8.7% (27/309) were adults (>49 years old) (Table 1). The children aged 1–5 years had the highest incidence of an *S. pneumoniae* infection compared with the younger and older children (≤1 and 6–12 years old) (*p* < 0.05), while the younger children displayed a higher incidence of infection than the older children (*p* < 0.05). Pneumonia was the most common disease in the children (78.7%, 222/282), while meningitis (37.0%, 10/27) was the most common disease in the adults (*p* < 0.05). These data suggest that children are the most susceptible population to a pneumococcal infection, while pneumonia and meningitis are the predominant types of pneumococcal diseases in children and adults, respectively.

**TABLE 1:** *S. pneumoniae*-infected diseases and susceptible populations.

		Age (years)						
		Child				Adult		
Disease	Case (*n*)	≤1	1–5	6–12	Total	49–65	>65	Total
URTI	38	3	24	7	34	0	4	4
Pneumonia	229	74	131	17	222^c^	3	4	7
Tympanitis	7	4	2	1	7	0	0	0
Bacteremia	23	4	8	5	17	3	3	6^d^
Meningitis	12	/	/	2	2	5	5	10^e^
Total	309	85^a^	165^b^	32	282	11	16	27

*URTI, upper respiratory tract infection.*

*^a^χ^2^ = 30.29, ^b^χ^2^ = 45.98, ^c^χ^2^ = 35.80, ^d^χ^2^ = 9.38, and ^e^χ^2^ = 87.12.*

*All the χ^2^-values were statistically significant (p < 0.05).*

### Predominant Serotypes in the *S. pneumoniae* Isolates

**FIGURE 1:**
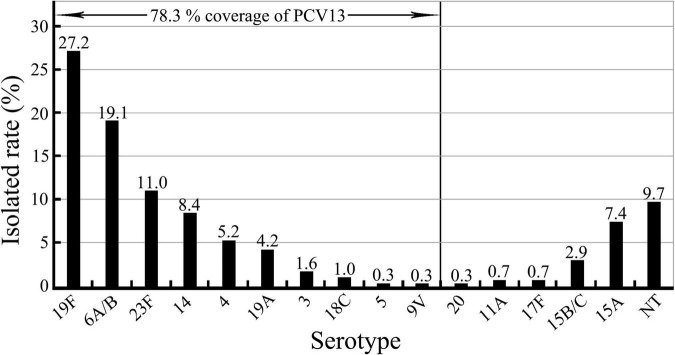
Serotypes in the 309 *S. pneumoniae* isolates and serotype coverage of pneumococcal vaccine PCV13.

All the 309 *S. pneumoniae* isolates were positive for the *S. pneumoniae*-specific 16S rRNA gene, and in 99.4% (307/309) of the isolates, the *cpsA* gene was detected. Of the 309 isolates, 90.3% (279/309) could be classified by PCR into fifteen serotypes (Table 2 and Supplementary Figure 1), while for 9.7% (30/309) of the isolates, a serotype could not be determined [non-typeable (NT)]. Among the fifteen identified serotypes, serotypes 19F (27.2%, 84/309) and 6A/B (19.1%, 59/309) were the most predominant (*p* < 0.05). Moreover, 23F, 14, 15A, and 4 were also as the common pneumococcal serotypes with a >5% incidence. More importantly, only 78.3% (242/309) of the 309 *S. pneumoniae* isolates belonged to thirteen serotypes covered by the 13-valent pneumococcal capsule conjugate vaccine (PCV13) mainly used in China for vaccination, while serotypes 15A (7.4%, 23/309) and 15B/C (2.9%, 9/309) were not included in this vaccine (Figure 1). These data show that 19F and 6A/B are the predominant pneumococcal serotypes prevailing in East China, while serotypes 15A and 15B/C should be added into the pneumococcal vaccine used in China.

**TABLE 2:** Serotypes of the 309 *S. pneumoniae* isolates.

Serotype	Isolate (*n*)	Proportion (%)
19F	84	27.2^a^
6A/B	59	19.1^b^
23F	34	11.0
14	26	8.4
15A	23	7.4
4	16	5.2
19A	13	4.2
15B/C	9	2.9
3	5	1.6
18C	3	1.0
11A	2	0.7
17F	2	0.7
5	1	0.3
9V	1	0.3
20	1	0.3
NT	30	9.7
Total	309	100

*^a^χ^2^ = 5.69 and ^b^χ^2^ = 7.91.*

*All the χ^2^-values were statistically significant (p < 0.05).*

### Correlation Between the *S. pneumoniae* Serotypes and Patient Ages and Diseases

The chi-square test and Fisher’s exact probability examination showed that serotypes 19F, 6A/B, 23F, 14, 15A, and 4, the predominant or common identified *S. pneumoniae* serotypes, caused a higher total infection rate in the children (≤1–12 years old) than in the adults (49–>65 years old) (*p* < 0.05) (Table 3). Furthermore, the children aged 1–5 years old had significantly higher positive rates of each of the predominant/common pneumococcal serotypes than the younger and older children (≤1 and 6–12 years old) (Table 3). On the contrary, these predominant/common pneumococcal serotypes displayed the highest incidence of pneumonia compared with the other four pneumococcal diseases (*p* < 0.05) (Table 4). These data suggest that children aged between 1 and 5 years are the most susceptible population to an infection by *S. pneumoniae*, and pneumonia is the most common pneumococcal disease.

**TABLE 3:** Correlation between predominant/common *S. pneumoniae* serotypes and patient ages.

		Serotype (*n*/%)					
Age (year)	Case (*n*)	19F	6A/B	23F	14	15A	4
≤1	85	32/37.6^a^	9/10.6	4/4.7	6/7.1	9/10.6	5/5.9
1–5	165	36/21.8	43/26.1^d^	19/11.5	18/10.9	13/7.9	8/4.8
6–12	32	10/31.3^b^	7/21.9^e^	4/12.5	2/6.3	1/3.1	1/3.1
49–65	7	1/14.3	0/0	3/42.9^f^	0/0	0/0	1/14.3
≥65	20	5/25.0^c^	0/0	4/20.0^g^	0/0	0/0	1/5.0
Total	309	84/27.2	59/19.1	34/11.0	26/8.4	23/7.4	16/5.2

*^a^χ^2^ = 22.15, ^b^χ^2^ = 12.84, ^c^χ^2^ = 6.21, ^d^χ^2^ = 12.16, ^e^χ^2^ = 6.25, ^f^χ^2^ = 60.17, and ^g^χ^2^ = 18.00.*

*All the χ^2^-values were statistically significant (p < 0.05).*

**TABLE 4:** Correlation between predominant/common *S. pneumoniae* serotypes and disease types.

		Serotype (*n*/%)					
Disease	Case (*n*)	19F	6A/B	23F	14	15A	4
URTI	38	12/31.6^a^	9/23.7^d^	4/10.5	0/0	2/5.3	3/7.9
Pneumonia	229	58/25.3	47/20.5	20/8.7	23/10.0	21/9.2	11/4.8
Tympanitis	7	5/71.4^b^	0/0	0/0	1/14.3	0/0	0/0
Bacteremia	23	7/30.4^c^	3/13.0	6/26.1^e^	2/8.7	0/0	1/4.3
Meningitis	12	2/16.7	0/0	4/33.3^f^	0/0	0/0	1/8.3
Total	309	84/27.2	59/19.1	34/11.0	26/8.4	23/7.4	16/5.2

*URTI, upper respiratory tract infection.*

*^a^χ^2^ = 9.18 ^b^χ^2^ = 66.27, ^c^χ^2^ = 7.19, ^d^χ^2^ = 6.54, ^e^χ^2^ = 16.51, and ^f^χ^2^ = 27.43.*

*All the χ^2^-values were statistically significant (p < 0.05).*

### Drug Resistance of the *S. pneumoniae* Isolates

**FIGURE 2:**
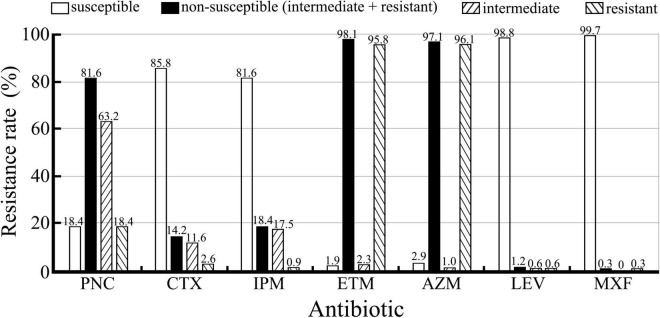
Resistance of the 309 *S. pneumoniae* isolates against seven antibiotics detected using the *E*-test. Non-susceptible are from the combination of intermediate and resistant.

The β-lactamase phenotype confirmatory test confirmed that all the 309 *S. pneumoniae* isolates did not produce β-lactamases. Drug resistance of the isolates against seven antibiotics is shown in Figure 2. The isolates displayed higher incidences of susceptibility to IPM, CTX, LEV, and MXF (81.6–99.7%, 252–308/309) but a low incidence of susceptibility to PNC (18.4%, 57/309). However, 81.6% (252/309) of the isolates were non-susceptible to PNC, including 63.2% (195/309) intermediate and 18.4% (57/309) resistant. To our surprise, the incidence of resistance to ETM (95.8%) and AZM (96.1%) was high. Furthermore, the analysis of the antibiotic resistance patterns among the *S. pneumoniae* isolates showed that 96.1% (297/309) of the isolates were multiple resistant with resistance against ETM+AZM (239/300, 79.7%) and PNC+ETM+AZM (47/300, 15.7%) being the most common multidrug resistance patterns (Table 5). In particular, the chi-square test showed that serogroups 19F, 6A/B, and 23F serotypes were closely correlated with PNC resistance (*p* < 0.05) (Table 6). These data suggest that the *S. pneumoniae* isolates prevailing in East China are commonly resistant to ETM and AZM, with ETM+AZM being the most dominant multidrug resistance pattern, and the predominant pneumococcal serotypes (19F, 6A/B, and 23F) are significantly correlated with PNC resistance.

**TABLE 5:** Multidrug resistance patterns of the *S. pneumoniae* isolates.

Resistance pattern	Isolate (*n*/%)
PNC	1/0.3
AZM	2/0.7
PNC+IPM	1/0.3
ETM+AZM	239/79.7^a^
PNC+ETM+AZM	47/15.7^b^
CTX+ETM+AZM	1/0.3
PNC+CTX+ETM+AZM	5/1.7
PNC+ETM+AZM+LEV	1/0.3
ETM+AZM+LEV+MXF	1/0.3
PNC+CTX+IPM+ETM+AZM	2/0.7
Total	300/100

*^a^χ^2^ = 246.29 and ^b^χ^2^ = 37.12.*

*All the χ^2^-values were statistically significant (p < 0.05).*

**TABLE 6:** β-lactam antibiotic susceptibility of prevalent *S. pneumoniae* serotypes.

Antibiotic	19F (*n* = 84)			6A/B (*n* = 59)			23F (*n* = 34)			14 (*n* = 26)			15A (*n* = 23)			4 (*n* = 16)		
	S	I	R	S	I	R	S	I	R	S	I	R	S	I	R	S	I	R
PNC	4	52^a^	28^b^	11	41^c^	7^d^	5	21^e^	8^f^	5	18^g^	3	5	17^h^	1	7	9	0
CTX	48	30	6	58	1	0	32	2	0	25	1	0	0	0	0	16	0	0
IPM	50	33	1	57	2	0	26	8	0	22	4	0	21	2	0	16	0	0

*S, susceptible; I, intermediate; R, resistant.*

*^a^χ^2^ = 11.53, ^b^χ^2^ = 17.85, ^c^χ^2^ = 59.15, ^d^χ^2^ = 6.17, ^e^χ^2^ = 23.72, ^f^χ^2^ = 9.07, ^g^χ^2^ = 6.58, and ^h^χ^2^ = 26.97.*

*All the χ^2^-values were statistically significant (p < 0.05).*

### Virulence Gene Distribution in the *S. pneumoniae* Isolates

**FIGURE 3:**
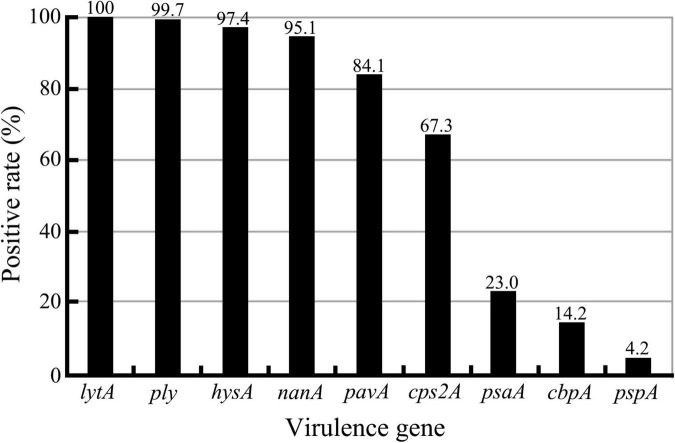
Positive rates of nine virulence genes in the 309 *S. pneumoniae* isolates detected by PCR.

Although all the nine virulence genes, namely, *cbpA*, *cps2A*, *hysA*, *lytA*, *nanA*, *pavA*, *ply*, *psaA*, and *pspA*, were detectable in this study (Supplementary Figure 2), there were large diversities in the positive rates for different virulence genes among the 309 *S. pneumoniae* isolates (Figure 3 and Tables 7, 8). All the isolates (309/309) were positive for the *lytA* gene, while the *nanA*, *hysA*, and *ply* genes were detected in 95.1% (294/309), 97.4% (301/309), and 99.7% (308/309) of the isolates, respectively. In contrast, the positive rates for the *pavA* and *cps2A* genes in the isolates were 84.1% (260/309) and 67.3% (208/309), respectively, while only 23.0% (71/309), 14.2% (44/309), and 4.2 (13/309) of the isolates contained the *psaA*, *cbpA*, and *pspA* genes, respectively. In particular, the chi-square test showed that the *pavA* and *psaA* genes were significantly correlated with pneumococcal bacteremia and meningitis, and serotype 23F displayed the highest positive rates of the *cps2A*, *psaA*, and *cbpA* genes (Tables 7, 8).

**TABLE 7:** Detection rates of *S. pneumoniae* virulence genes in different diseases.

		Virulence gene (*n*/%)								
Disease	Case (*n*)	*lytA*	*ply*	*hysA*	*nanA*	*pavA*	*cps2A*	*psaA*	*cbpA*	*pspA*
URTI	38	38/100	38/100	37/97.4	37/97.4	33/78.9^a^	25/65.8	9/23.7	4/10.5	1/2.6
Pneumonia	229	229/100	228/99.6	223/97.4	215/93.9	189/82.5^b^	152/66.4	47/20.5	33/14.4	11/4.8
Tympanitis	7	7/100	7/100	7/100	7/100	4/57.1	4/57.1	4/57.1^e^	0/0	1/14.3
Bacteremia	23	23/100	23/100	22/95.7	23/100	22/95.7^c^	18/78.3	7/30.4^f^	5/21.7^h^	0/0
Meningitis	12	12/100	12/100	12/100	12/100	12/100^d^	9/75.0	4/33.3^g^	2/16.7	0/0
Total	309	309/100	308/99.7	301/97.4	294/95.1	260/84.1	208/67.3	71/23.0	44/14.2	13/4.2

*^a^χ^2^ = 6.53, ^b^χ^2^ = 8.99, ^c^χ^2^ = 19.00, ^d^χ^2^ = 23.55, ^e^χ^2^ = 12.80, ^f^χ^2^ = 4.00, ^g^χ^2^ = 6.38, and ^h^χ^2^ = 7.81.*

*All the χ^2^-values were statistically significant (p < 0.05).*

**TABLE 8:** Detection rates of *S. pneumoniae* virulence genes in predominant/common serotypes.

		Virulence gene (*n*/%)								
Serotype	Case (*n*)	*lytA*	*ply*	*hysA*	*nanA*	*pavA*	*cps2A*	*psaA*	*cbpA*	*pspA*
19F	84	84/100	84/100	79/94.0	83/98.8	68/81.0	48/57.1	27/32.1	11/13.1	0/0
6A/B	59	59/100	59/100	59/100	55/93.2	50/84.7	35/59.3	0/0	8/13.6	6/3.4
23F	34	34/100	34/100	32/94.1	34/100	28/82.4	29/85.3^a^	13/38.2^b^	7/20.6^c^	0/0
14	26	26/100	26/100	25/96.2	24/92.3	20/76.9	19/73.1	3/11.5	3/11.5	1/3.8
15A	23	23/100	23/100	23/100	21/91.3	21/91.3	15/65.2	4/17.4	1/4.3	2/8.7
4	16	16/100	15/93.8	16/100	16/100	14/87.5	12/75.0	2/12.5	3/18.8	1/6.3
Total	242	242/100	241/99.6	234/96.7	233/96.3	201/74.8	158/65.3	49/20.2	33/13.6	10/4.1

*^a^χ^2^ = 11.04, ^b^χ^2^ = 29.76, and ^c^χ^2^ = 5.23.*

*All the χ^2^-values were statistically significant (p < 0.05).*

## Discussion

Previous epidemiological investigation data consistently revealed that children are much more susceptible to *S. pneumoniae* than adults, and pneumonia is the most common pneumococcal disease in children (Erlichman et al., 2017; GBD 2016 Lower Respiratory Infections Collaborators, 2018; Wang et al., 2020). Our study also showed that 91.3% (282/309) of the 309 *S. pneumoniae*-infected patients were children, and 78.7% (222/282) of them suffered from pneumonia. Although our study contained much fewer *S. pneumoniae*-infected adult patients (8.7%, 27/309), with 37.0% (10/27), the incidence of meningitis in the adult patients was strikingly high. Since meningitis is usually considered as a highly lethal disease, doctors must pay more attention to the adult patients infected with *S. pneumoniae*.

*Streptococcus pneumoniae* strains can be classified into many different serotypes according to their antigenic diversity of their capsular polysaccharides (Jauneikaite et al., 2015; Zafar et al., 2017; Ganaie et al., 2020; Joshi et al., 2020). However, there are differences in the predominant and common serotypes of *S. pneumoniae* in different areas and counties, probably due to different vaccine types and vaccination rates (Klugman and Rodgers, 2021). Recent epidemiological studies reported the predominant or common serotypes of *S. pneumoniae* isolates prevailing in the different areas and adjacent countries of China. 19F, 6B, 23F, and 19A were the common serotypes observed among the *S. pneumoniae* isolates in Western China, while 19F, 19A, and 23F were the predominant serotypes among the *S. pneumoniae* isolates in North China (Zhao et al., 2020; Liang et al., 2021; Zhou et al., 2022). In the different areas of Southwest China, 19F and 19A were the predominant pneumococcal serotypes (Shen et al., 2017; Ma et al., 2021; Yan et al., 2021). In addition, serotypes 12F, 3, 23A, 19A, 10A, 6C, and 22F and serotypes 3, 19A, and 11A were the commonly found among the *S. pneumoniae* isolates from Japan and South Korea, respectively (Park et al., 2019; Heo et al., 2020; Yanagihara et al., 2021). Furthermore, 23F, 19F, 19A, and 14 were the predominant pneumococcal serotypes found in Thailand and Malaysia (Wangirapan et al., 2020; Dzaraly et al., 2021). In India, 14, 1, and 19F acted as the predominant serotypes of *S. pneumoniae* (Singh et al., 2017). However, in our study covering East China, 19F and 6A/B were the predominant serotypes, while 23F, 14, 15A, and 4 were the other common serotypes observed, which are distinct from the predominant and common pneumococcal serotypes found in the other areas of China and in the other counties in Asia. More importantly, PCV13, a 13-valent pneumococcal capsule conjugate vaccine mainly used in China (Wang et al., 2021), appears to only provide a 78.3% coverage of the *S. pneumoniae* isolates characterized in this study, while the commonly found serotypes 15A (7.4%, 23/309) and 15B/C (2.9%, 9/309) are not included in PCV13. These data suggest that the predominant and common *S. pneumoniae* serotypes from East China display a regional specificity, and that capsular polysaccharide antigens from pneumococcal 15A and 15B/C serotypes with a total positive rate of 10.4% should be included in the multivalent capsule conjugate vaccine.

β-Lactam antibiotics serve as the first-line therapy choice for the treatment of pneumococcal diseases. However, β-lactam antibiotic resistance rates of *S. pneumoniae* isolates from different areas and countries have a large diversity. For example, PNC and CTX resistance rates among the *S. pneumoniae* isolates from Western China, North China, and Southwest China were 1.2–79.9% and 0.5–28.6% (Shen et al., 2017; Zhao et al., 2020; Liang et al., 2021; Yan et al., 2021; Zhou et al., 2022), while the PNC resistance rates among the *S. pneumoniae* isolates from East Asia (Japan and South Korea), Southeast Asia (Thailand and Malaysia), India, United States, Europe, and Latin America were 1.7–22.8%, 4–83.1%, 10%, 10.8%, 19.4%, and 68.6%, respectively (Mendes et al., 2016; Yahiaoui et al., 2016; Singh et al., 2017; Park et al., 2019; Moreno et al., 2020; Wangirapan et al., 2020; Dzaraly et al., 2021; Yanagihara et al., 2021). In this study, the PNC non-susceptible rate among the 309 *S. pneumoniae* isolates was as high as 81.6% (63.2% intermediate and 18.4% resistant), but the CTX non-susceptible rate was only 14.2% (11.6% intermediate and 2.6% resistant). The ETM and AZM resistance rates among the 309 *S. pneumoniae* isolates were 95.8 and 96.1%, which are similar to those from Western China (94.3%), North China (95–98.2%), and Southwest China (99.1%) (Zhao et al., 2020; Liang et al., 2021; Yan et al., 2021; Zhou et al., 2022) but significantly higher than those from Southeast Asia (38.5–42.0%), India (37.0%), United States (37.1%), and Latin America (43.2%) (Mendes et al., 2016; Singh et al., 2017; Moreno et al., 2020; Wangirapan et al., 2020; Dzaraly et al., 2021). In addition, 96.1% of the 309 *S. pneumoniae* isolates in this study showed different multidrug resistance patterns, which is also similar to those from Western China (93.8%) (Liang et al., 2021), but significantly higher than those from South Korea (46.2%), Malaysia (18.0%), and Latin America (44.3%) (Park et al., 2019; Moreno et al., 2020; Dzaraly et al., 2021). More importantly, *S. pneumoniae* serotypes 19F, 6A/B, and 23F displayed a significant correlation with PNC resistance. These data suggest that the *S. pneumoniae* isolates from East China are commonly resistant to PNC, ETM, and AZM, and that the PNC resistance may contribute to the prevalence of pneumococcal serotypes 19F, 6A/B, and 23F.

*Streptococcus pneumoniae* has no endotoxin (i.e., LPS) and typical exotoxins, but its capsule plays a crucial role in pathogenesis through antiphagocytosis activity and benefiting colonization (Nelson et al., 2007; van der Poll and Opal, 2009; Brissac et al., 2021). In this study, only two of the 309 *S. pneumoniae* isolates did not contain the *cpsA* gene, a capsule formation-determining factor (Dias et al., 2007; Coskun-Ari et al., 2012), highlighting the importance of the capsule in pneumococcal pathogenesis. Of the nine pneumococcal virulence genes analyzed in this study, all the *S. pneumoniae* isolates were positive for the *lytA* gene, while the *nanA*, *hysA*, and *ply* genes were detectable in 95.1–99.7% of the isolates, which is similar to the positive detection rates (91.2 and 96.2%) of the *ply* and *lytA* genes in the 419 *S. pneumoniae* isolates from Western China and the positive rates (93.1, 97.1, and 100%) of the *nanA*, *cbpA*, and *ply* genes in the 102 *S. pneumoniae* isolates from Southwest China (Yan et al., 2019; Liang et al., 2021). Previous studies also confirmed that the *lytA* gene, encoding the *S. pneumoniae* autolysin, had high specificity and sensitivity for pneumococcal detection (Ramos-Sevillano et al., 2015; Afshar et al., 2020). Therefore, the product of the *lytA* gene can be used to identify *S. pneumoniae* and act as a candidate antigen of pneumococcal vaccines. Although the *hysA* and *ply* genes showed the 97.4 and 99.7% positive rates among the *S. pneumoniae* isolates, respectively, their products, a secretory hyaluronidase and an intracytoplasmic pneumolysin (Marion et al., 2012; Greene et al., 2015; Rai et al., 2016), are unsuitable for vaccine development. The product of the *nanA* gene (95.1% positive rates) is a surface neuraminidase of *S. pneumoniae* and can therefore also be used as a potential antigen for development of novel pneumococcal vaccines.

## Data Availability Statement

The original contributions presented in the study are included in the article/Supplementary Material, further inquiries can be directed to the corresponding author.

## Ethics Statement

Written informed consent was obtained from the individual(s), and minor(s)’ legal guardian/next of kin, for the publication of any potentially identifiable images or data included in this article.

## Author Contributions

A-HS and Y-YH obtained the funds. A-HS and JY conceived and designed the experiments. L-DH and M-JY performed the experiments and analyzed the data. K-YJ performed the passage of *S. pneumoniae* isolates. L-DH, M-JY, JY, and A-HS wrote the manuscript. All authors reviewed the manuscript.

## Conflict of Interest

The authors declare that the research was conducted in the absence of any commercial or financial relationships that could be construed as a potential conflict of interest.

## Publisher’s Note

All claims expressed in this article are solely those of the authors and do not necessarily represent those of their affiliated organizations, or those of the publisher, the editors and the reviewers. Any product that may be evaluated in this article, or claim that may be made by its manufacturer, is not guaranteed or endorsed by the publisher.
