# Supplementary material for: Molecular Characterization of Predominant Serotypes, Drug Resistance, and Virulence Genes of Streptococcus pneumoniae Isolates From East China
Source: Front Microbiol. 2022 Jun 1;13:892364. doi: 10.3389/fmicb.2022.892364 (PMC9198556; doi:10.3389/fmicb.2022.892364)
Supplement: Supplementary file 1 [file Data_Sheet_1.doc]

**Molecular Characterization of Predominant Serotypes, Drug Resistance and Virulence of *Streptococcus pneumoniae* Isolates from East China**

Li-Dan Huang, Mei-Juan Yang, Yan-Ying Huang, Ke-Yi Jiang, Jie Yan, Ai-Hua Sun

**SUPPLEMENTARY MATERIAL**

**MATERIALS AND METHODS**

**Serotyping of *Streptococcus pneumoniae* isolates**

The serotypes of 309 *S. pneumoniae* isolates were identified using PCR as previously reported (Pai et al., 2006; Dias et al., 2007; Da Gloria-Carvalho, et al., 2010). Using 100 ng DNA of each of the genomic DNA samples as the template, separate PCRs were performed using a High-Fidelity Ex-Taq PCR Kit (TaKaRa, China) and deferent pairs of serotyping primers (Table S1).

**Detection of Virulence Genes in *S. pneumoniae* Isolates**

The nine virulence genes in each of the 309 *S. pneumoniae* isolates, *cbpA*, *cps2A*, *hysA*, *lytA*, *nanA*, *pavA*, *ply*, *psaA* and *pspA* genes for pneumococcal adherence, colonization, invasion and survival (Holmes et al., 2001; Romero-Steiner et al., 2003; Marion et al., 2012; Croney et al., 2013; Mellro et al., 2014; Rai et al., 2016; Wren et al., 2017; Subramanian et al., 2019; Zhao et al., 2019) were detected by separate PCRs using a High-Fidelity Ex-Taq PCR Kit (TaKaRa) and deferent pairs of primers (Table S2).

**RESULTS**

**Serotypes in the *S. pneumoniae* Isolates**

In the 309 *S. pneumoniae* isolates, 90.3% (279/309) could be classified into fifteen serotypes by the PCRs (Figure S1) but 9.7% (30/309) could not be determined for their serotypes (nontypeable, NT).

**Virulence Gene Distribution in the *S. pneumoniae* Isolates**

All the nine virulence genes, *cbpA*, *cps2A*, *hysA*, *lytA*, *nanA*, *pavA*, *ply*, *psaA* and *pspA*, could be detectable from *S. pneumoniae* isolates in this study (Figure S2), but there were large diversities in the positive rates of different virulence genes in the pneumococcal isolates.

**REFERENCES**

Croney, C.M., Nahm, M.H., Juhn, S.K., Briles, D.E., Crain, M.J. Invasive and noninvasive (2013). *Streptococcus pneumoniae* capsule and surface protein diversity following the use of a conjugate vaccine. *Clin. Vaccine. Immunol.* 20, 1711-1718. doi:10.1128/CVI.00381-13

Da. Gloria-Carvalho, M., Pimenta, F.C., Jackson, D., Roundtree, A., Ahmad, Y., Millar, E.V., et al. (2010). Revisiting pneumococcal carriage by use of broth enrichment and PCR techniques for enhanced detection of carriage and serotypes. *J. Clin. Microbiol.* 48, 1611-1618. doi: 10.1128/JCM.02243-09

Dias, C.A., Teixeira, L.M., Carvalho, M.D.G., Beall, B. (2007). Sequential multiplex PCR for determining capsular serotypes of pneumococci recovered from Brazilian children. *J. Med. Microbiol.* 56, 1185-1188. doi: 10.1099/jmm.0.47347-0

Holmes, A.R., McNab, R., Millsap, K.W., Rohde, M., Hammerschmidt, S., Mawdsley, J.L, et al. (2001). The *pavA* gene of *Streptococcus pneumoniae* encodes a fibronectin-binding protein that is essential for virulence. *Mol. Microbiol.* 41, 1395-1408. doi:10.1046/j.1365-2958.2001.02610.x

Marion, C., Stewart, J.M., Tazi, M.F., Burnaugh, A.M., Linke, C.M., Woodiga, S.A., et al. (2012). *Streptococcus pneumoniae* can utilize multiple sources of hyaluronic acid for growth. *Infect. Immun.* 80, 1390-1398. doi: 10.1128/IAI.05756-11

Mellro, P., Sandalova, T., Kikhney, A., Vilaplana, F., Hesek, D., Lee, M., et al. (2014). Structural and functional insights into peptidoglycan access for the lytic amidase LytA of *Streptococcus pneumoniae*. *mBio.* 5, e01120-13. doi:10.1128/mBio.01120-13

Pai, R., Gertz, R.E., Beall, B. (2006). Sequential multiplex PCR approach for determining capsular serotypes of *Streptococcus pneumoniae* isolates. *J. Clin. Microbiol.* 44, 124-131. doi:[10.1128/JCM.44.1.124-131.2006](https://doi.org/10.1128/jcm.44.1.124-131.2006)

Rai, P., He, F., Kwang, J., Engelward, B.P., Chow, V.T. (2016). Pneumococcal pneumolysin induces DNA damage and cell cycle arrest. *Sci. Rep.* 6, 22972-22983. doi:[10.1038/srep22972](https://doi.org/10.1038/srep22972)

Romero-Steiner, S., Pilishvili, T., Sampson, J.S., Johnson, S.E., Stinson, A., Carlone, G.M., et al. (2003). Inhibition of pneumococcal adherence to human nasopharyngeal epithelial cells by anti-PsaA antibodies. *Clin. Diagn. Lab. Immunol.* 10, 246-251. doi:[10.1128/cdli.10.2.246-251.2003](https://doi.org/10.1128/cdli.10.2.246-251.2003)

Subramanian, K., Henriques-Normark, B., Normark, S. (2019). Emerging concepts in the pathogenesis of the *Streptococcus pneumoniae*: From nasopharyngeal colonizer to intracellular pathogen. *Cell Microbiol.* 21, e13077-13086. doi: 10.1111/cmi.13077

Wren, J.T., Blevins, L.K., Pang, B., Basu-Roy, A., Oliver, M.B., Reimche, J.L., et al. (2017). Pneumococcal neuraminidase a (NanA) promotes biofilm formation and synergizes with influenza a virus in nasal colonization and middle ear infection. *Infect. Immun.* 85, e01044-16. doi: 10.1128/IAI.01044-16

Zhao, W.T., Pan, F., Wang, B.J., Sun, Y., Zhang, T.D., Shi, Y.Y., et al. (2019). [Epidemiology characteristics of *Streptococcus pneumoniae* from children with pneumonia in Shanghai: a retrospective study.](https://pubmed.ncbi.nlm.nih.gov/31380301/) *Front. Cell Infect. Microbiol.* 9, 258-267. doi:10.3389/fcimb.2019.00258

**FIGURE LEGENDS**

FIGURE S1 Amplification fragments from different *S. pneumoniae* serotypes by PCR

Lane M: DNA marker. Lane 1: blank control. Lanes 2 and 3: amplification fragments of 16S rRNA gene (217 bp) and *cpsA* gene (160 bp) as the controls. Lanes 4-18: amplification fragments of 19F (304 bp), 6A/B (250 bp), 23F (384 bp), 14 (189 bp), 15A (436 bp), 4 (430 bp), 19A (478 bp), 15B/C (496 bp), 3 (371 bp), 18C (573 bp), 11A (463 bp), 17F (693 bp), 5 (362 bp)，9V (753 bp) and 20 (514 bp) serotypes, respectively.

FIGURE S2 Amplification fragments from different *S. pneumoniae* virulence genes by PCR

Lane M: DNA marker. Lane 1: blank control. Lane 3: amplification fragment of 16S rRNA gene (217 bp) as the control. Lanes 4-12: amplification fragments of *lytA* (308 bp), *ply* (329 bp), *hysA* (240 bp), *nanA* (500 bp), *pavA* (540 bp), *cps2A* (517 bp), *psaA* (838 bp), *cbpA* (570 bp) and *pspA* (663 bp) genes, respectively.

TABLE S1 Primers used in this study for serotyping of *S. pneumoniae* isolates

| Serotype | Primer sequence (5’ to 3’) | Product (bp) |
| --- | --- | --- |
| 1 | F: CTCTATAGAATGGAGTATATAAACTATGGTTA | 280 |
|  | R: CCAAAGAAAATACTAACATTATCACAATATTGCC |  |
| 2 | F: TATCCCAGTTCAATATTTCTCCACTACACC | 290 |
|  | R: ACACAAAATATAGGCAGAGAGAGACTACT |  |
| 3 | F: ATGGTGTGATTTCTCCTAGATTGGAAAGTAG | 371 |
|  | R: CTTCTCCAATTGCTTACCAAGTGCAATAACG |  |
| 4 | F: CTGTTACTTGTTCTGGACTCTCGATAATTGG | 430 |
|  | R: GCCCACTCCTGTTAAAATCCTACCCGCATTG |  |
| 5 | F: ATACCTACACAACTTCTGATTATGCCTTTGTG | 362 |
|  | R: GCTCGATAAACATAATCAATATTTGAAAAAGTATG |  |
| 6A/B | F: AATTTGTATTTTATTCATGCCTATATCTGG | 250 |
|  | R: TTAGCGGAGATAATTTAAAATGATGACTA |  |
| 6C/D | F: CATTTTAGTGAAGTTGGCGGTGGAGTT | 727 |
|  | R: AGCTTCGAAGCCCATACTCTTCAATTA |  |
| 7F | F: CCTACGGGAGGATATAAAATTATTTTTGAG | 826 |
|  | R: CAAATACACCACTATAGGCTGTTGAGACTAAC |  |
| 8 | F: GATGCCATGAATCAAGCAGTGGCTATAAATC | 294 |
|  | R: ATCCTCGTGTATAATTTCAGGTATGCCACC |  |
| 9N/L | F: GAACTGAATAAGTCAGATTTAATCAGC | 516 |
|  | R: ACCAAGATCTGACGGGCTAATCAAT |  |
| 9V | F: CTTCGTTAGTTAAAATTCTAAATTTTTCTAAG | 753 |
|  | R: GTCCCAATACCAGTCCTTGCAACACAAG |  |
| 10A | F: GGTGTAGATTTACCATTAGTGTCGGCAGAC | 628 |
|  | R: GAATTTCTTCTTTAAGATTCGGATATTTCTC |  |
| 11A | F: GGACATGTTCAGGTGATTTCCCAATATAGTG | 463 |
|  | R: GATTATGAGTGTAATTTATTCCAACTTCTCCC |  |
| 12F | F: GCAACAAACGGCGTGAAAGTAGTTG | 376 |
|  | R: CAAGATGAATATCACTACCAATAACAAAAC |  |
| 13 | F: TACTAAGGTAATCTCTGGAAATCGAAAGG | 655 |
|  | R: CTCATGCATTTTATTAACCGCTTTTTGTTC |  |
| 14 | F: GAAATGTTACTTGGCGCAGGTGTCAGAATT | 189 |
|  | R: GCCAATACTTCTTAGTCTCTCAGATGAAT |  |
| 15A | F: ATTAGTACAGCTGCTGGAATATCTCTTC | 434 |
|  | R: GATCTAGTGAACGTACTATTCCAAAC |  |
| 15B/C | F: TTGGAATTTTTTAATTAGTGGCTTACCTA | 496 |
|  | R: CATCCGCTTATTAATTGAAGTAATCTGAACC |  |
| 16F | F: GAATTTTTCAGGCGTGGGTGTTAAAAG | 717 |
|  | R: CAGCATATAGCACCGCTAAGCAAATA |  |
| 17F | F: TTCGTGATGATAATTCCAATGATCAAACAAGAG | 693 |
|  | R: GATGTAACAAATTTGTAGCGACTAAGGTCTGC |  |
| 18C | F: CTTAATAGCTCTCATTATTCTTTTTTTAAGCC | 573 |
|  | R: TTATCTGTAAACCATATCAGCATCTGAAAC |  |
| 19A | F: GTTAGTCCTGTTTTAGATTTATTTGGTGATGT | 478 |
|  | R: GAGCAGTCAATAAGATGAGACGATAGTTAG |  |
| 19F | F: GTTAAGATTGCTGATCGATTAATTGATATCC | 304 |
|  | R: GTAATATGTCTTTAGGGCGTTTATGGCGATAG |  |
| 20 | F: GAGCAAGAGTTTTTCACCTGACAGCGAGAAG | 514 |
|  | R: CTAAATTCCTGTAATTTAGCTAAAACTCTTATC |  |
| 21 | F: CTATGGTTATTTCAACTCAATCGTCACC | 192 |
|  | R: GGCAAACTCAGACATAGTATAGCATAG |  |
| 22F | F: GAGTATAGCCAGATTATGGCAGTTTTATTGTC | 643 |
|  | R: CTCCAGCACTTGCGCTGGAAACAACAGACAAC |  |
| 23A | F: TATTCTAGCAAGTGACGAAGATGCG | 722 |
|  | R: CCAACATGCTTAAAAACGCTGCTTTAC |  |
| 23B | F: CCACAATTAGCGCTATATTCATTCAATCG | 199 |
|  | R: GTCCACGCTGAATAAAATGAAGCTCCG |  |
| 23F | F: GTAACAGTTGCTGTAGAGGGAATTGGCTTTTC | 384 |
|  | R:CACAACACCTAACACACGATGGCTATATGATTC |  |
| 31 | F: GGAAGTTTTCAAGGATATGATAGTGGTGGTGC | 701 |
|  | R: CCGAATAATATATTCAATATATTCCTACTC |  |
| 33F | F: GAAGGCAATCAATGTGATTGTGTCGCG | 338 |
|  | R: CTTCAAAATGAAGATTATAGTACCCTTCTAC |  |
| 34 | F: GCTTTTGTAAGAGGAGATTATTTTCACCCAAC | 408 |
|  | R: CAATCCGACTAAGTCTTCAGTAAAAAACTTTAC |  |
| 35B | F: GATAAGTCTGTTGTGGAGACTTAAAAAGAATG | 677 |
|  | R: CTTTCCAGATAATTACAGGTATTCCTGAAGCAAG |  |
| 39 | F: TCATTGTATTAACCCTATGCTTTATTGGTG | 98 |
|  | R: GAGTATCTCCATTGTATTGAAATCTACCAA |  |
| *cpsA* | F: GCAGTACAGCAGTTTGTTGGACTGACC | 160 |
|  | R: GAATATTTTCATTATCAGTCCCAGTC |  |
| 16S rRNA | F: CTGTGGCTTAACCATAGTAG | 217 |
|  | R: CTAGCACTCATCGTTTACA |  |

F: forward primers. R: reverse primers. 16S rRNA and *cpsA* genes were used as the controls.

TABLE S2 Primers used in this study for detecting virulence genes of *S. pneumoniae*

| Gene | Primer sequence (5’ to 3’) | Product (bp) |
| --- | --- | --- |
| *cbpA* | F: CGAGGGCACAATTAAGCAAGCA | 570 |
|  | R: CGATCTGTCTTGATGTTTTCTAACCT |  |
| *cps2A* | F: GTAGAAGCTCCTAAGACGTCTA | 517 |
|  | R:GAAGGATAGCCACAATCACCT |  |
| *hysA* | F: GCGGAAGAAACGACTACGAA | 240 |
|  | R: AGGGCTTGAGATAGTGATAG |  |
| *lytA* | F: CAACCGTACAGAATGAAGCGG | 308 |
|  | R: TTATTCGTGCAATACTCGTGCG |  |
| *nanA* | F: ATAGACGTGCGCAAAATACAGAATCA | 500 |
|  | R: GTCGAACTCCAAGCCAATAACTCCT |  |
| *pavA* | F: TGCAAATCAGGCGGGAGAGCCTT | 540 |
|  | R: TGGATTTTCTCCCGTTGTCTTCT |  |
| *ply* | F: ATTTCTGTAACAGCTACCAACGA | 329 |
|  | R: GAATTCCCTGTCTTTTCAAAGTC |  |
| *psaA* | F: CATAGACTAGAACAAGAGCTCAAA | 838 |
|  | R: CTACATTATTGTTTTCTTCAGCAG |  |
| *pspA* | F: AGGTTTCCGTGCTCCTCTTC | 663 |
|  | R:CCGTTAGCGTTGAGGTAATACCA |  |
| 16S rRNA | F: CTGTGGCTTAACCATAGTAG | 217 |
|  | R: CTAGCACTCATCGTTTACA |  |

F: forward primers. R: reverse primers. 16S rRNA gene was used as the control.

**FIGURE LEGENDS**

FIGURE S1 Amplification fragments from different *S. pneumoniae* serotypes by PCR

Lane M: DNA marker. Lane 1: blank control. Lanes 2 and 3: amplification fragments of 16S rRNA gene (217 bp) and *cpsA* gene (160 bp) as the controls. Lanes 4-18: amplification fragments of 19F (304 bp), 6A/B (250 bp), 23F (384 bp), 14 (189 bp), 15A (436 bp), 4 (430 bp), 19A (478 bp), 15B/C (496 bp), 3 (371 bp), 18C (573 bp), 11A (463 bp), 17F (693 bp), 5 (362 bp)，9V (753 bp) and 20 (514 bp) serotypes, respectively.

FIGURE S2 Amplification fragments from different *S. pneumoniae* virulence genes by PCR

Lane M: DNA marker. Lane 1: blank control. Lane 3: amplification fragment of 16S rRNA gene (217 bp) as the control. Lanes 4-12: amplification fragments of *lytA* (308 bp), *ply* (329 bp), *hysA* (240 bp), *nanA* (500 bp), *pavA* (540 bp), *cps2A* (517 bp), *psaA* (838 bp), *cbpA* (570 bp) and *pspA* (663 bp) genes, respectively.
