# Supplementary figures and images for: Molecular Characterization of Predominant Serotypes, Drug Resistance, and Virulence Genes of Streptococcus pneumoniae Isolates From East China
Source: Front Microbiol. 2022 Jun 1;13:892364. doi: 10.3389/fmicb.2022.892364 (PMC9198556; doi:10.3389/fmicb.2022.892364)

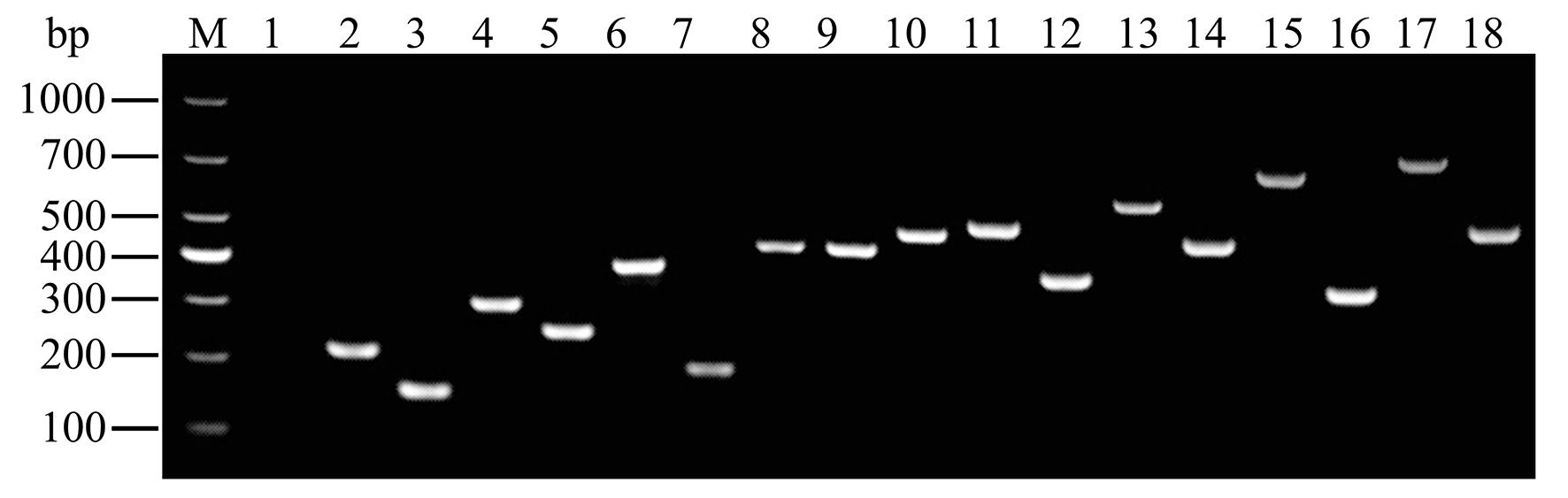

Supplement: Supplementary file 2 [file Image_1.TIF]

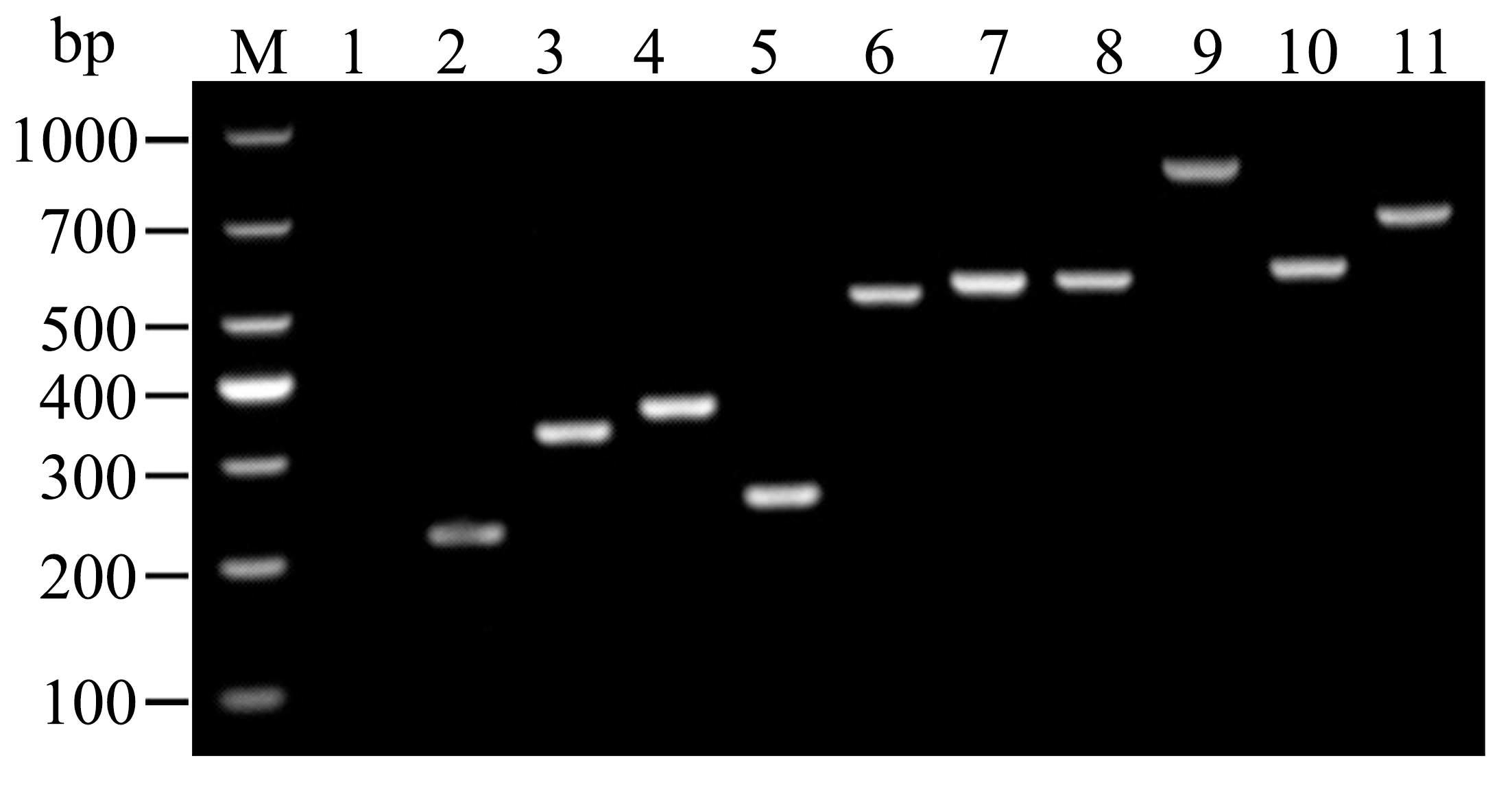

Supplement: Supplementary file 3 [file Image_2.TIF]
